# Performances of automated digital imaging of Gram-stained slides with on-screen reading against manual microscopy

**DOI:** 10.1007/s10096-021-04233-2

**Published:** 2021-05-08

**Authors:** Adrien Fischer, Nouria Azam, Lara Rasga, Valérie Barras, Manuela Tangomo, Gesuele Renzi, Nicolas Vuilleumier, Jacques Schrenzel, Abdessalam Cherkaoui

**Affiliations:** 1 Bacteriology Laboratory, Division of Laboratory Medicine, Department of Diagnostics, Geneva University Hospitals, 4 rue Gabrielle-Perret-Gentil, 1205 Geneva, Switzerland; 2 Division of Laboratory Medicine, Department of Diagnostics, Geneva University Hospitals and Faculty of Medicine, Geneva, Switzerland; 3 Genomic Research Laboratory, Division of Infectious Diseases, Department of Medicine, Geneva University Hospitals and Faculty of Medicine, Geneva, Switzerland

**Keywords:** Gram stain, Manual microscopy, Automated digital imaging, On-screen reading, Digitalization

## Abstract

The objective of this study was to evaluate the performances of the automated digital imaging of Gram-stained slides against manual microscopy. Four hundred forty-three identified Gram-stained slides were included in this study. When both methods agreed, we considered the results as correct, and no further examination was carried out. Whenever the methods gave discrepant results, we reviewed the digital images and the glass slides by manual microscopy to avoid incorrectly read smears. The final result was a consensus of multiple independent reader interpretations. Among the 443 slides analyzed in this study, 101 (22.8%) showed discrepant results between the compared methods. The rates of discrepant results according to the specimen types were 5.7% (9/157) for positive blood cultures, 42% (60/142) for respiratory tract specimens, and 22% (32/144) for sterile site specimens. After a subsequent review of the discrepant slides, the final rate of discrepancies dropped to 7.0% (31/443). The overall agreement between the compared methods and the culture results reached 78% (345/443) and 79% (349/443) for manual microscopy and automated digital imaging, respectively. According to culture results, the specificity for automated digital imaging and manual microscopy were 90.8% and 87.7% respectively. In contrast, sensitivity was 84.1% for the two compared methods. The discrepant results were mostly encountered with microorganism morphologies of rare occurrence. The results reported in this study emphasize that on-screen reading is challenging, since the recognition of morphologies on-screen can appear different as compared to routine manual microscopy. Monitoring of Gram stain errors, which is facilitated by automated digital imaging, remains crucial for the quality control of reported Gram stain results.

## Introduction

By any measure, this decade has been outstanding in the history of automation in clinical microbiology. Automation enabled not only to customize each analytical step but also to force the laboratory managers to concentrate all the pre-analytical steps onto a unique physical interface that has become the entry door to all further analytical activities of conventional bacteriology. Two automated systems are currently available for clinical specimens streaking and slides preparation: Inoculated media are loaded onto conveyors for transfer to automated incubators where cultures are imaged with high-resolution digital pictures at pre-defined times. Direct consequences of total laboratory automation can be measured as improved productivity, traceability, quality, and reduced turn-around times [1–6]. But despite the implementation of these novel technologies, some traditional techniques (e.g., Gram stain) continue to bear an important role in the diagnostic process. The Gram-stained smears remain important as a pre-analytical indicator of respiratory tract specimen quality (e.g., sputum), for presumptive etiologic diagnosis, to guide empirical therapy and to indicate the presence of mixed aerobic and anaerobic infections. The Gram stain has therefore been a cornerstone for clinical bacteriology laboratories for over a century, despite the subjectivity of the results interpretation (highly operator-dependent) and the manual nature of the staining process. The interpretation of Gram stain results continues to be labor-intensive, time-consuming, and strongly dependent on the quality of the samples. To face the increasing workload in clinical microbiology laboratories, automated slide scanning and imaging might provide several advantages and adequately complement manual testing. Nonetheless, many technical challenges should be overcome before Gram-stain automation can be systematically deployed in bacteriology. For instance, the quality of the staining remains strongly affected by the smear preparation (markedly different for a thick sputum or a biopsy versus for a blood culture or a body fluid). Therefore, Gram-stained smears can display tremendously variable and heterogeneous background staining, which can obviously affect the algorithm that may target areas without bacteria and miss the most relevant microscopic zones.

The overarching objective of this study was to assess the performances of automated digital imaging of Gram-stained slides with on-screen reading against manual microscopy. Automated digital imaging was performed by the Metafer slide scanning platform that permits scanning, digitalization, and archiving of slides automatically, even in a batch mode.

## Materials and methods

### Slide collection and workup

A total of 443 identified Gram-stained slides from positive blood cultures (*n* = 157), respiratory tract specimens (*n* = 142), and sterile site specimens (*n* = 144) were collected in the clinical bacteriology laboratory of Geneva University Hospitals between February and June 2020. All the slides included in this study were prepared by the Copan WASP® during the routine clinical workup. Importantly, the slides were chosen without any preselection (e.g., staining quality, abundance, or identity of the microorganisms), and they were not pre-screened by automated digital imaging in order to ideally capture the variability of routine specimens. One hundred additional slides, encompassing all the specimen types analyzed in this study, were used during the training period to validate and evaluate the different parameters of the Metafer slide scanning and the imaging platform, according to the manufacturer’s instructions (MetaSystems Hard & Software GmbH, Altlussheim, Germany). These slides were not included in the subsequent study period. Slides from respiratory tract and sterile site specimens were stained using a manual method. In contrast, all slides from positive blood cultures were stained using the PREVI® Color Gram (BioMérieux, Marcy L’Etoile, France). The slides analysis for each workflow was performed by four experienced laboratory technologists and two clinical microbiologists by rotating after a training period, in order to avoid any learning bias. Importantly, all were blinded from the results obtained using the other method.

### Culture diagnostic workup

All specimens included in this study were processed on the WASPLab following the protocols previously published [1–3].

### Discrepant results

**Fig. 1:**
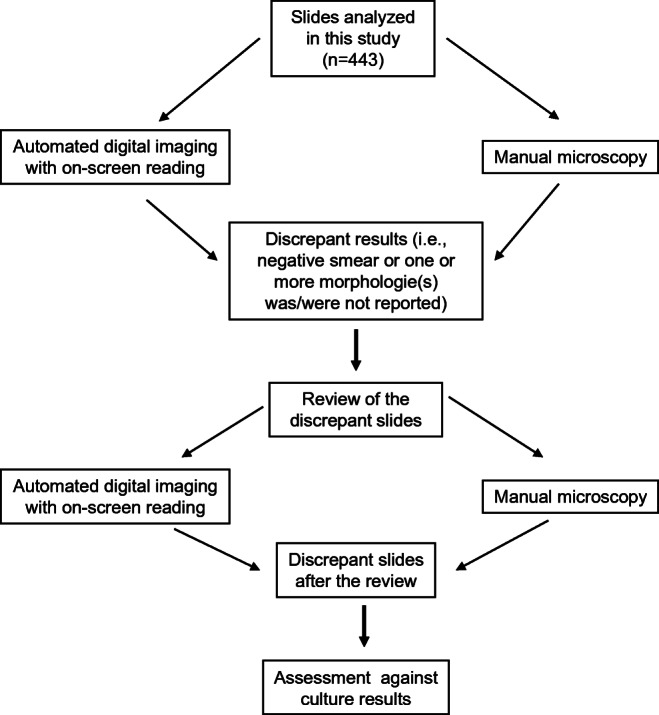
Algorithm of results assessment between automated digital imaging with on-screen reading and manual microscopy

The results of automated digital imaging with on-screen reading were compared to the manual microscopy. When both methods agreed, we considered the results as correct, and no further examination was carried out. Whenever the methods gave discrepant results (i.e., negative smear or one or more morphologies was/were not reported), we reviewed the digital images and the glass slides by manual microscopy to avoid incorrectly read smears. For the remaining discrepant slides, the Gram strain results were assessed against culture results (Fig. 1). The final result was a consensus of multiple independent reader interpretations.

### Metafer slide scanning and imaging platform

In this study, we used a commercial off-the-shelf software. All the 443 slides included in this study plus the 100 slides used during the training period were imaged without coverslips using a Metafer slide scanning and imaging platform with a 160-slide-capacity automated slide loader equipped with × 10 and × 40 magnification objectives (Carl Zeiss AG, Oberkochen, Germany) and automatic random access of slides. The × 10 magnification was used for collecting images spanning the whole slide. Collected images were then stitched together to create a single digital picture of the Gram smear. Right after, 20 images were taken with seven focal planes for each such picture using a × 40 oil immersion lens magnification for defined areas, according to the manufacturer’s instructions. On-screen reading of such digital images was performed using the Metafer 5 software.

## Results

Among the 443 slides analyzed in this study, 101 (22.8%) showed discrepant results between manual microscopy and automated digital imaging using on-screen reading. The rates of discrepant results according to the specimen types were 5.7% (9/157) for positive blood cultures, 42% (60/142) for respiratory tract specimens, and 22% (32/144) for sterile site specimens. A large part of incorrectly read slides were those where one or more microorganism morphologies was/were not reported. After a subsequent review of these discrepant slides, the rate of discrepant results dropped drastically to 7.0% (31/443). More specifically, the rates according to the specimen types became 0.6% (1/157) for positive blood cultures, 15% (21/142) for respiratory tract specimens, and 6.3% (9/144) for sterile site specimens (Table 1). As depicted in Table 2, the commonly missed microorganism morphologies by manual microscopy versus automated digital imaging with on-screen reading were Gram-negative rods (39% (12/31) versus 23% (7/31)), Gram-positive rods (6.5% (2/31) versus 19.4% (6/31)), and Gram-positive cocci (6.5% (2/31) versus 16.1% (5/31)) respectively. In contrast, less commonly missed microorganism morphologies were Gram-negative cocci ((0% versus 6.5% (2/31)) and yeasts ((0% versus 3.2% (1/31)). The discrepant results were seen mostly in microorganism morphologies of rare occurrence.

**Table 1:** Rates of discrepant results broken by specimen types

Specimen types	Total number of specimens	Number of discrepant results	Number of discrepant results after review of the digital images	Number of discrepant results after review of the slides by manual microscopy	Final agreement including reviewed slides (%)
Blood cultures	157	9	1	1	99.4
Respiratory tract specimens	142	60	46	21	85.2
Sterile site specimens	144	32	15	9	93.8
Total	443	101	62	31	93

**Table 2:** Analysis of discrepant results

Specimen types	Manual microscopy result	Digital images result	Culture result
Bronchoalveolar lavage	Gram-positive cocci, and Gram-negative rods	Gram-positive cocci	Oropharyngeal flora
Bronchoalveolar lavage	Gram-positive cocci	Gram-positive cocci, and Gram-negative rods	Oropharyngeal flora
Bronchoalveolar lavage	Gram-positive cocci, Gram-negative rods, and Gram-negative cocci	Gram-positive cocci, and Gram-negative rods	Oropharyngeal flora
Bronchoaspiration	Gram-positive cocci	Negative smear	Oropharyngeal flora
Bronchoaspiration	Gram-positive cocci, and Gram-positive rods	Gram-positive cocci	Oropharyngeal flora
Bronchoaspiration	Gram-positive cocci, and Gram-positive rods	Gram-positive cocci, Gram-positive rods, and Gram-negative rods	Oropharyngeal flora
Tracheal aspiration	Gram-positive cocci, Gram-negative rods, and yeast	Yeast	*Candida glabrata*, oropharyngeal flora
Tracheal aspiration	Gram-positive cocci	Gram-negative rods	*Enterococcus faecium*
Sputum	Gram-positive cocci	Negative smear	*Pseudomonas aeruginosa*, and oropharyngeal flora
Sputum	Gram-positive cocci, and Gram-negative rods	Gram-positive cocci	*Enterobacterales*, and oropharyngeal flora
Sputum	Gram-positive cocci, and Gram-positive rods	Gram-positive cocci, and Gram-negative rods	*Candida albicans, Enterobacteriales*, and oropharyngeal flora
Sputum	Gram-positive cocci, and yeast	Gram-positive cocci, and Gram-negative rods	*Staphylococcus aureus, Candida albican*s, and oropharyngeal flora
Sputum	Gram-positive cocci, and Gram-negative rods	Gram-positive cocci, Gram-positive rods, and Gram-negative rods	*Staphylococcus aureus, Enterobacterales*, and oropharyngeal flora
Sputum	Gram-positive cocci, and yeast	Gram-positive cocci, Gram-negative rods, and yeast	*Pseudomonas aeruginosa, Candida albicans,* and oropharyngeal flora
Sputum	Gram-positive cocci, and Gram-positive rods	Gram-positive cocci	Oropharyngeal flora
Sputum	Gram-negative cocci, and yeast	Gram-negative rods, and yeast	*Candida albicans*, and Enterobacterales
Sputum	Gram-positive cocci, Gram-positive rods, Gram-negative rods, and yeast	Gram-positive cocci, Gram-negative rods, and yeast	*Haemophilus influenzae*, and oropharyngeal flora
Sputum	Gram-positive cocci, Gram-negative rods, and yeast	Gram-positive cocci, and yeast	*Candida glabrata*, and oropharyngeal flora
Sputum	Gram-positive cocci, and Gram-positive rods	Gram-positive cocci, Gram-positive rods, and Gram-negative rods	Oropharyngeal flora
Sputum	Gram-positive cocci, and yeast	Gram-positive cocci, Gram-negative rods, and yeast	Enterobacterales, and oropharyngeal flora
Sputum	Gram-positive cocci, and Gram-positive rods	Gram-positive cocci, and Gram-negative rods	*Pseudomonas aeruginosa, Enterobacterale*s, and oropharyngeal flora
Oropharyngeal smear	Gram-positive cocci, and Gram-negative rods	Gram-positive cocci, Gram-positive rods, and Gram-negative rods	Oropharyngeal flora
Oropharyngeal smear	Gram-positive cocci	Gram-positive cocci, and Gram-negative rods	Oropharyngeal flora
Deep wound smear	Gram-positive cocci, Gram-positive rods, and Gram-negative rods	Gram-positive cocci	*Streptococcus anginosus, Prevotella buccae*, *Candida albicans*, and Gram-positive flora
Deep wound smear	Gram-positive cocci	Gram-positive cocci, Gram-negative rods	*Staphylococcus aureus,* and *Streptococcus agalactiae*
Deep wound smear	Negative smear	Gram-positive cocci	Gram positive flora
Deep wound smear	Negative smear	Gram-negative rods	*Morganella morganii*
Deep wound smear	Gram-positive rods	Gram-positive cocci, and Gram-positive rods	*Staphylococcus aureus*, and *Bacillus simplex*
Biopsy	Gram-negative rods	Negative smear	Negative culture
Deep wound smear	Gram-positive cocci, and yeast	Gram-positive cocci, Gram-negative rods, and yeast	Mixed flora, and *Candida albicans*
Positive blood culture	Gram-negative rods	Negative smear	*Fusobacterium nucleatum*

According to culture results, 6.5% (2/31) and 9.7% (3/31) of the remaining discrepant results were smear negative and culture positive for manual microscopy and automated digital imaging with on-screen reading, respectively. Additionally, for 32.3% (10/31) of the remaining discrepant results, only oropharyngeal flora was observed on culture media. The assessment of the 31 discrepant reviewed slides against culture highlighted that 42% (13/31) and 38.7% (12/31) were discordant with the culture results for manual microscopy and automated digital imaging with on-screen reading, respectively (Table 2). The performances of the two compared methods according to culture results are depicted in Table 3.

**Table 3:** The performances of the two compared methods according to culture results

Manual microscopy	% Sensitivity	% Specificity	% PPV	% NPV
Blood cultures	100	100	100	100
Respiratory tract specimens	75	100	100	17.1
Sterile site specimens	58.2	88.7	89.8	55.3
Total	81.3	90.8	98	46.8
Automated digital imaging	% Sensitivity	% Specificity	% PPV	% NPV
Blood cultures	98.7	100	100	75
Respiratory tract specimens	80.2	83.3	98.9	17.9
Sterile site specimens	51.6	83	83.9	50
Total	80.7	84.6	96.7	44.4
Performances after a subsequent review of the discrepant slides				
Manual microscopy	% sensitivity	% specificity	% PPV	% NPV
Blood cultures	100	100	100	100
Respiratory tract specimens	81.9	83.3	99	19.2
Sterile site specimens	60.4	86.8	88.7	56.1
Total	84.1	87.7	97.4	50
Automated digital imaging	% sensitivity	% specificity	% PPV	% NPV
Blood cultures	99.3	100	100	85.7
Respiratory tract specimens	81	83.3	98.9	18.5
Sterile site specimens	62.6	90.6	91.9	58.5
Total	84.1	90.8	98	50.9

*NPV* negative predictive value, *PPV* positive predictive value

## Discussion

The Gram stain belongs to those tests routinely performed in clinical microbiology laboratories that are prone to variability and to the subjectivity of their interpretation [7, 8]. In many cases, Gram stain errors can have a significant clinical impact, especially for sterile site specimens and blood cultures, underlining why clinical microbiology laboratories perform frequent quality controls to monitor the correlation between Gram stain results and cultures. Several reports have examined Gram stain errors rates and highlighted the major drivers of such errors [9–12]. Moreover, the interpretation of Gram stain results remains labor-intensive, time-consuming, highly subjective, and strongly dependent on the specimen types and on the smear quality. Nowadays, the steadily increasing workload for clinical analyses challenges clinical microbiology laboratories, facing the divergent needs to improve quality, productivity, and turn-around times while simultaneously rationalizing the laboratory technologists’ workforce. Some of these challenges can therefore further impact the Gram stain errors rates by precluding a daily review of smears that showed discrepant results with cultures, and decrease the ongoing quality control. To decrease the rates of discrepant results for sterile fluid samples, an additional slide is systematically performed and stained with acridine orange in our laboratory, even if the sensitivity of Gram and acridine orange-staining remains suboptimal compared to culture in the rapid diagnosis of septic arthritis [13].

Using automated digital imaging with on-screen reading, we assessed the overall slide classification accuracy on the 443 Gram-stained smears which were previously classified by manually microscopy. The overall agreement between both methods was 77%. However, after a subsequent review of the discrepant slides, the overall agreement reached 93%. The rate of discrepant results was markedly different between the three specimen types included in this study. Specifically, the agreements between the compared methods according to the specimen types were 99.4%, 85.2%, and 93.8% for positive blood cultures, respiratory tract specimens, and sterile site specimens, respectively. Despite the fact that not all observed bacteria in a specimen may be recovered in culture due to either a lack of viability or overgrowth by a more predominant organism(s), the overall agreement between the compared methods and the culture results reached 78% (345/443) and 79% (349/443) for manual microscopy and automated digital imaging using on-screen reading, respectively.

In the context of a multicenter evaluation of Gram stain error study, Samuel et al. reported that 24% of discrepant Gram strain results were linked to interpretation errors by the technologists, across the different study sites [11, 12]. Based on the observations made during our study, specific factors were highlighted as the cause of Gram stain errors using the automated digital imaging with on-screen reading: 1) the recognition of microorganism morphologies on-screen can appear very different and more challenging to identify as compared to routine manual microscopy, 2) the nature of smear preparation, and 3) the thick smears with high cellular content are also especially challenging. To mitigate such errors, smears with inadequate material should be repeated in order to increase the number and the quality of fields examined in addition to the double review of the smears. While this approach might reduce error rates, the logistics appears arduous. Double review of smears can therefore be routinely performed only for a subset of specimens, focusing for example only on blood cultures and sterile fluids. Finally, the reporting and categorization of Gram stain errors by types of error and technologists can help revealing patterns, for targeted review or for additional training.

## Conclusion

While the results reported in this study were not surprising given the subjective nature of Gram stains, they emphasize that on-screen reading is challenging even to experienced professionals; the laboratory technologists should therefore benefit from additional and specific training coupled to performance assessment. Additionally, the monitoring of Gram-stain errors, which is facilitated by automated digital imaging, represents a crucial step in the process of improving the quality of Gram stain results. Automated digital imaging of Gram-stained slides permits improved diagnostic workflow by facilitating the slides review and the exchange of information and by building educational picture libraries containing challenging smears that are the source of the most frequent errors. Finally, on-screen reading of digital images affords huge practical and ergonomic advantages as compared to the tedious manual microscopy and constitutes a useful complement to manual microscopy.

## References

[1] Cherkaoui A, Renzi G, Viollet A, Fleischmann M, Metral-Boffod L, Dominguez-Amado D, Vuilleumier N, Schrenzel J (2020) Implementation of the WASPLab and first year achievements within a university hospital. Eur J Clin Microbiol Infect Dis 10.1007/s10096-020-03872-1 32248509

[2] Cherkaoui A, Renzi G, Vuilleumier N, Schrenzel J (2019). Copan WASPLab automation significantly reduces incubation times and allows earlier culture readings. Clin Microbiol Infect.

[3] Cherkaoui A, Renzi G, Azam N, Schorderet D, Vuilleumier N, Schrenzel J (2020) Rapid identification by MALDI-TOF/MS and antimicrobial disk diffusion susceptibility testing for positive blood cultures after a short incubation on the WASPLab. Eur J Clin Microbiol Infect Dis 10.1007/s10096-020-03817-8 31965365

[4] Cherkaoui A, Renzi G, Charretier Y, Blanc DS, Vuilleumier N, Schrenzel J (2019). Automated incubation and digital image analysis of chromogenic media using Copan WASPLab enables rapid detection of vancomycin-resistant enterococcus. Front Cell Infect Microbiol.

[5] Bailey AL, Burnham CD (2019). Reducing the time between inoculation and first-read of urine cultures using total lab automation significantly reduces turn-around-time of positive culture results with minimal loss of first-read sensitivity. Eur J Clin Microbiol Infect Dis.

[6] Burckhardt I, Last K, Zimmermann S (2019). Shorter incubation times for detecting multi-drug resistant bacteria in patient samples: defining early imaging time points using growth kinetics and total laboratory automation. Ann Lab Med.

[7] Nagendra S, Bourbeau P, Brecher S, Dunne M, LaRocco M, Doern G (2001). Sampling variability in the microbiological evaluation of expectorated sputa and endotracheal aspirates. J Clin Microbiol.

[8] Musher DM, Montoya R, Wanahita A (2004). Diagnostic value of microscopic examination of gram-stained sputum and sputum cultures in patients with bacteremic pneumococcal pneumonia. Clin Infect Dis.

[9] Rand KH, Tillan M (2006). Errors in interpretation of gram stains from positive blood cultures. Am J Clin Pathol.

[10] Goodyear N, Kim S, Reeves M, Astion ML (2006). A 2-year study of gram stain competency assessment in 40 clinical laboratories. Am J Clin Pathol.

[11] Samuel LP, Balada-Llasat JM, Harrington A, Cavagnolo R (2016). Multicenter assessment of gram stain error rates. J Clin Microbiol.

[12] Samuel LP, Balada-Llasat JM, Harrington A, Cavagnolo R (2016). Correction for Samuel et al., multicenter assessment of gram stain error rates. J Clin Microbiol.

[13] Cunningham G, Seghrouchni K, Ruffieux E, Vaudaux P, Gayet-Ageron A, Cherkaoui A, Godinho E, Lew D, Hoffmeyer P, Uckay I (2014). Gram and acridine orange staining for diagnosis of septic arthritis in different patient populations. Int Orthop.

